# The Effect of *Aspergillus oryzae* (Koji) on the Physicochemical and Sensory Characteristics and Satiating Capacity of Angus Beef

**DOI:** 10.3390/foods15081296

**Published:** 2026-04-09

**Authors:** Cristina Filip, Victoria Ancuta Nyulas, Maria Czinege, Amalia Puscas, Amelia Tero-Vescan, Ioan Costa, Florina Ruta

**Affiliations:** 1 Department of Biochemistry, George Emil Palade University of Medicine, Pharmacy, Science and Technology of Târgu Mureș, Gheorghe Marinescu Street No 38, 540136 Târgu Mureș, Romania; cristina.filip@umfst.ro (C.F.); amalia.puscas@umfst.ro (A.P.); amelia.tero-vescan@umfst.ro (A.T.-V.); 2 Department of Medical Informatics and Biostatistics, George Emil Palade University of Medicine, Pharmacy, Science and Technology of Târgu Mureș, Gheorghe Marinescu Street No 38, 540136 Târgu Mureș, Romania; 3 Department of Community Nutrition and Food Safety, George Emil Palade University of Medicine, Pharmacy, Science and Technology of Târgu Mureș, Gheorghe Marinescu Street No 38, 540136 Târgu Mureș, Romania; maria.czinege@umfst.ro (M.C.); florina.ruta@umfst.ro (F.R.); 4 Department of Food Innovation, Fermentation and Enzymatic Meat Alternatives, EnzymMeat^®^ & Delectatum Research Unit, Conservation Funghi Association (NGO), 540151 Târgu Mureș, Romania; condimentedelectatum@gmail.com

**Keywords:** beef meat, *Aspergillus oryzae*, sensory characteristics, satiating capacity

## Abstract

Considering the increasing consumer demand for natural meat tenderization methods, this study explores the potential of *Aspergillus oryzae* (Koji) to enhance beef quality. The aim of this study was to evaluate the enzymatic effect of *Aspergillus oryzae* (*A. oryzae*) on the physicochemical and sensory characteristics, as well as the perception of satiety, in Angus beef. Two distinct anatomical cuts, the neck and the round, were subjected to enzymatic aging using four different Koji-based mixtures. Parameters such as water content, thermal preparation (grilling) loss, expressible moisture, and pH were determined, supplemented by sensory analysis and a satiety test. Compared to untreated or traditionally marinated samples (Teriyaki sauce), Koji-treated samples exhibited lower grilling loss and improved texture. Sensory analysis highlighted a more intense flavor profile and increased acceptability of the enzymatically treated products. The satiety test indicated a predominantly positive perception of postprandial fullness, with negative ratings being rare and exclusive to the control group. These results support the potential of *A. oryzae* as a natural alternative for optimizing the technological and sensory quality of red meat, contributing to a favorable consumer experience, including satiety perception.

## 1. Introduction

In a broader context, fermented foods and the microorganisms involved in the fermentation process are increasingly analyzed not only for their technological and sensory properties but also in relation to their impact on human health. Diet is recognized as a major factor in modulating the gut microbiota, and its imbalances (dysbiosis) have been associated with various conditions, including pathologies seemingly weakly linked to nutrition, such as recurrent urinary tract infections or irritable bowel syndrome. Research indicates that changes in microbiota composition can influence the inflammatory response, amino acid metabolism, and intestinal barrier function, highlighting the indirect yet relevant role of diet and fermented foods in maintaining general health and recovery after illness [1,2].

In this context, fermented products obtained with food-safe microorganisms, such as *Aspergillus oryzae*, are gaining increasing importance not only as functional ingredients in food processing but also as components of a diversified diet capable of influencing the nutritional quality and acceptability of animal-based foods.

However, despite its extensive use in the fermentation industry, scientific data regarding the impact of koji on red meat, particularly on Angus beef, remain limited. Given the significance of physicochemical characteristics, sensory profiles, and perceived satiety, investigating how *Aspergillus oryzae* influences these aspects may contribute significantly to the development of sustainable meat processing methods [3].

Koji (*Aspergillus oryzae*) is a filamentous fungus traditionally employed in Asian food fermentations such as the production of sake, miso, and soy sauce owing to its intense enzymatic activity. This fungus produces a complex suite of proteolytic and amylolytic enzymes capable of degrading proteins, starches, and other macromolecular compounds, thereby enhancing digestibility and flavor development in fermented products [4]. Beyond its traditional use, Koji (*Aspergillus oryzae*) has expanded into the field of meat processing as a biological agent for tenderization. Due to its protease activity, it can efficiently break down myofibrillar proteins and connective tissue, significantly improving texture and palatability [5,6].

Despite the documented effects of Koji on meat tenderness, little is known about how these enzymatic modifications influence post-consumption physiological responses. We hypothesize that applying *Aspergillus oryzae* to Angus beef not only improves physicochemical properties and texture through targeted proteolysis but also enhances sensory perception and satiety. Therefore, this study evaluates the effects of four Koji-based treatments on two beef cuts (neck and round) and assesses their impact on consumer satiety

## 2. Materials and Methods

### 2.1. Sample Preparation for Physicochemical and Sensory Determinations

Two muscle groups were utilized: the round (specifically the top sirloin/rump—denoted as R) and the neck/chuck (specifically the top blade/chuck—denoted as N). These were sourced from Aberdeen Angus cattle (Black Angus variety), raised on a specialized farm in the Măgherani area (Mureș County), a region in Transylvania, Romania, renowned for extensive beef cattle farming. For each muscle group, both aged samples and control samples were prepared.

The Koji used in this study was a commercial product based on *Aspergillus oryzae* (powder form), supplied by a local producer from Sovata (Mureș County, Sovata, Romania), obtained from a selected strain (Strain III). The protease activity of the *Aspergillus oryzae* preparation was approximately 800 U/g, as specified by the supplier. This value is consistent with the typical range reported for commercial *A. oryzae* protease preparations used in food fermentation and meat tenderization (generally 300–1500 U/g) [7,8,9].

The aged samples consisted of four types, obtained by treating the meat with: Koji-Mix-Shiitake (KM), Shio-Koji (KS), Koji incubated at 32 °C (RK 32), and Koji incubated at 25 °C (RK 25). Prior to application, the enzymatic powder was hydrated for 2 h at 22 °C. For the control samples (RC and NC), Teriyaki sauce was used for maceration (Table 1).

The Koji-based enzymatic mixture was applied manually to the surface of the meat through uniform massaging until the pieces were completely coated. The hydrated paste was distributed in a continuous layer, and the massaging process facilitated direct contact between the enzymes and the muscle fibers, thereby optimizing the diffusion of proteases into the tissue.

All samples were seasoned identically. For this purpose, a standard spice blend was used, containing sweet paprika, tomato powder, parsley leaves, oregano leaves, ground mixed peppercorns, non-iodized salt, chili, onion, rosemary, basil, and corn starch.

Thermal preparation (TP) of the samples was done by grilling until reaching an internal temperature of 57–59 °C, corresponding to a medium degree of cooking. The core temperature was monitored using a digital probe thermometer, and the TP process was terminated immediately upon reaching the target range to ensure the comparability of the samples during sensory analysis.

Two muscle groups were used in this study: the round (top sirloin/rump, coded as R) and the neck/chuck (top blade/chuck, coded as N). Both were sourced from Aberdeen Angus cattle raised on a specialized farm in the Măgherani area (Mureș County, Romania). According to the supplier’s specifications for Aberdeen Angus beef, the round (R) samples contained approximately 20–22% protein, 4–6% fat, 72–74% moisture, and 1.0–1.2% ash, while the neck/chuck (N) samples contained 18–20% protein, 8–12% fat, 68–70% moisture, and 1.0–1.2% ash. These values were included to ensure transparency regarding the raw material composition.

A commercial *Aspergillus oryzae* (Koji) powder (Strain III), supplied by a local producer from Sovata (Mureș County, Romania), was used for all enzymatic treatments. For all variants, the Koji paste was applied at a ratio of 2 g Koji powder per 100 g of meat (2% *w*/*w*). Prior to application, Koji powder was hydrated using a 1.5:1 water-to-powder ratio (*w*/*w*) to obtain a uniform enzymatic paste. For the Shio-Koji variant, the mixture contained 25% fine non-iodized salt relative to the Koji powder mass.

Four Koji-based treatments were prepared (Table 1):

1. Koji-Mix-Shiitake (KM)

Koji powder was blended with shiitake mushroom powder (70:30 *w*/*w*) and hydrated at 22 °C for 3 h. The paste was applied uniformly to the meat surface, and samples were aged under refrigeration (2–4 °C) for 72 h.

2. Shio-Koji (KS)

Koji powder was mixed with 25% non-iodized salt and hydrated at 22 °C for 3 h. The paste was applied evenly to the meat, followed by 72 h of refrigerated enzymatic maturation. Excess paste was removed by gentle rinsing before cooking.

3. RK32 (Koji activated at 32 °C)

Koji powder was hydrated at 32–33 °C for 2 h to activate thermophilic proteases. The paste was applied in two thin layers, with a 30 min interval between applications, then aged under refrigeration for 72 h.

4. RK25 (Koji activated at 25 °C)

Koji powder was hydrated at 22 °C for 5 h, promoting slower enzymatic activation. The paste was applied in a single uniform layer, and samples were aged under refrigeration for 72 h.

Control samples (RC and NC) were marinated using Teriyaki sauce under identical conditions (72 h at 2–4 °C). All samples were seasoned identically using a standardized spice blend (sweet paprika, tomato powder, parsley, oregano, mixed peppercorns, non-iodized salt, chili, onion, rosemary, basil, and corn starch) to ensure comparability across treatments.

### 2.2. Determination of Physicochemical Properties

The moisture content of the samples was determined using the dry-heating method until a constant weight was achieved according to the AOAC official method [10]. The moisture content was calculated using the following formula [4]:

Moisture content % = [(W1 − W2)/W1] × 100,

where W1 and W2 represent the initial weight and the final weight after drying, respectively.

Weight losses due to thermal treatment were calculated by weighing the samples before and after TP, using the formula [4]:

TP loss % = [(W1 − W2)/W1] × 100,

where W1 and W2 represent the initial weight and the final weight after thermal treatment, respectively.

For pH determination, a Hanna Edge Dedicated pH/ORP digital pH meter with a measurement range of 0–14 was used. Two grams of each sample were homogenized with 18 mL of distilled water for 1 min. The results obtained represent the arithmetic mean of six successive measurements [4].

The meat samples were weighed before and after being subjected to enzymatic treatment or maceration (in the case of the control samples) to determine the uptake of curing. The following formula was used for this determination [4]:

Uptake of curing % = [(W2 − W1)/W1] × 100,

where W1 represents the initial weight of the sample and W2 represents the weight of the sample after the enzymatic treatment or maceration.

Expressible moisture (EM) represents the amount of liquid, primarily water, released from a protein system when an external force is applied through pressure or centrifugation. It serves as an indicator of water-holding capacity (WHC), reflecting the volume of “free water” that can be extracted from the tissues under mechanical action. The ability of the samples to retain moisture was determined using 1 g of sample, which was placed into conical centrifuge tubes and centrifuged at 3000 rpm for 10 min at 4 °C. The results were calculated using the following formula [4]:

EM % = [(W1 − W2)/W1] × 100,

where W1 represents the initial weight of the sample before centrifugation and W2 represents the weight of the sample after centrifugation.

### 2.3. Evaluation of Sensory Characteristics

A total of 126 participants, including undergraduate and graduate students from the Nutrition and Dietetics program at the George Emil Palade University of Medicine, Pharmacy, Science, and Technology of Târgu Mureș, took part in the sensory analysis. All participants possessed foundational knowledge of sensory testing. The participants tasted samples of uniform size immediately after thermal preparation.

Sensory analysis was conducted to confirm the effects of the enzymatic process on the meat and to determine the degree of consumer acceptability. For this analysis, a 9-point hedonic scale was employed, which is a standardized and widely used method in sensory science. The following sensory parameters were evaluated: appearance, odor, taste, color, juiciness, tenderness, and overall preference. The results can be linked with the physicochemical changes induced by koji treatment and consumer-perceived quality, thereby supporting conclusions on the potential of koji-based enzymatic marination to enhance beef flavor. The sensory evaluation followed a repeated-measures design, as all panelists evaluated all treatment variants. Panelist identity was included as a random effect in the statistical model to account for within-subject correlation, while treatment was treated as a fixed effect. This design allowed the comparison of treatment effects while controlling for individual variability in perception.

### 2.4. Thermal Preparation (TP)

Thermal preparation (TP) was carried out by grilling the meat using an electric contact grill (OptiGrill+ XL, model GC722D, Tefal, Groupe SEB, Rumilly, France) equipped with flat cast-aluminum plates, preheated for 7–8 min at medium-high power, reaching a stable surface temperature of 200–220 °C. All grilling procedures were performed in the kitchen facilities of the Nutrition Centre of the George Emil Palade University of Medicine, Pharmacy, Science, and Technology of Târgu Mureș. To ensure strict standardization and avoid variability in heat transfer, the beef samples were grilled individually, each piece being placed on the preheated grill and monitored separately.

The grilling process was divided into three phases:

Initial phase: 1.5–2 min at 200–220 °C, with 10 g of butter added per slice; weighed using a Kern EMB 200-2 analytical kitchen scale (precision ± 0.01 g) to ensure accurate and standardized fat addition across all samples.

Intermediate phase: 3–4 min at 160–180 °C, turning the meat every 1.5 min.

Final phase: until the internal core temperature reached 57–59 °C.

The core temperature was monitored using a digital probe thermometer (Hanna Instruments, Checktemp^®^ 1, model HI-98509, accuracy ± 0.3 °C; Hanna Instruments, Woonsocket, RI, USA)). After grilling, samples were rested for 4–5 min before portioning.

### 2.5. Evaluation of Satiating Capacity

A total of 17 participants, including undergraduate and graduate students from the Nutrition and Dietetics program at the George Emil Palade University of Medicine, Pharmacy, Science, and Technology of Târgu Mureș, participated in the satiety analysis.

A satiety test was conducted to compare two beef variants: one enzymatically treated with koji (designated the TASTE Group) and one marinated with teriyaki sauce (designated the Control Group). Visual Analog Scales (VAS) for satiety were utilized as a method frequently employed in nutritional studies to evaluate subjective sensations such as hunger, fullness, and the desire to eat. This scale has been validated in clinical and nutritional studies for measuring dietary responses [11].

Additionally, an extended post-consumption VAS-hedonic hybrid scale (Martin) was used. This scale combines taste evaluation with satiety perception and re-consumption intent and is typically applied to understand consumer behavior in studies focused on innovative products, including alternative or fermented meats [12].

For this part of the study, 17 participants were recruited from the George Emil Palade University of Medicine, Pharmacy, Science, and Technology of Târgu Mureș. Inclusion criteria for the satiety test required participants to be aged between 18 and 60 years, healthy with no chronic pathologies, and following an omnivorous diet. Participants were informed that the study involved the consumption and evaluation of Angus beef marinated with different types of curing agents, prepared both with and without *Aspergillus oryzae*. All participants signed an informed consent form prior to the start of the experiment. Ethical approval for this study was granted by the Ethics Committee of the University (No. 3535 of 30 December 2024).

The meals were prepared at the kitchen facilities of the Nutrition Centre of the University. Prior to the test, participants were instructed to consume breakfast and to drink only water until the evaluation took place. At 12 PM, each participant consumed a meat portion that weighed 100 g prior to TP. The questionnaires were completed 30 min after finishing the meal. Each type of meat was consumed on separate days to prevent sensory fatigue or carry-over effects.

Study participants were aged between 18 and 60 years, presented no chronic pathologies, and followed an omnivorous dietary pattern. Subjects were informed that the study involved the consumption and evaluation of Angus beef marinated with various enzymatic treatments. All participants signed an informed consent form prior to the commencement of the experiment.

### 2.6. Statistical Analysis

All data were analyzed using IBM SPSS Statistics 26.0 (IBM Corp., Armonk, NY, USA). Physicochemical parameters, sensory scores, and satiety values were expressed as mean ± standard deviation (SD). Normality was verified using the Shapiro–Wilk test [13], and homogeneity of variances was assessed using Levene’s test.

Because each participant evaluated all treatments, sensory and satiety data were analyzed using a repeated-measures ANOVA, followed by Bonferroni-adjusted post hoc tests to identify pairwise differences. Physicochemical parameters were analyzed using one-way ANOVA with treatment as the fixed factor.

Categorical satiety classifications (low, medium, high) were analyzed using the Chi-square test, while continuous VAS scores were analyzed using repeated-measures ANOVA. Statistical significance was set at *p* < 0.05 for all analyses.

## 3. Results

### 3.1. Physicochemical Determinations

The analysis of the six replicates for each sample revealed that the minimum TP loss was observed in the RK25 (16.66 ± 0.45%) and NK25 (15.51%) groups. Conversely, the maximum marinade uptake (uptake of curing) was recorded for the RKS (5.61%) and NKS (4.80%) samples (Table 2).

One-way ANOVA revealed significant differences among the four sample groups (Round–Koji, Round–Control, Neck–Koji, Neck–Control) for all physicochemical parameters (*p* < 0.05). Post hoc pairwise comparisons with Bonferroni correction showed that Koji-treated samples differed significantly from their respective controls for water content, cooking loss, enzymatic uptake, and expressible moisture (*p* < 0.05). Differences between anatomical regions (Round vs. Neck) were also statistically significant for most parameters. Statistically significant differences are indicated in Table 2.

#### 3.1.1. Water Content, TP Loss, and pH

*Water Content (%).* Higher moisture values indicate superior juiciness retention. The analysis of the six replicates for each sample showed that the round (RC: 49.71%) has a higher water content compared to the neck (NC: 45.74%). The NK32 and NK25 variants (Koji powder activated at 25 °C and 32 °C, respectively) maintained a high moisture content exceeding 55%, suggesting efficient hydration and potentially increased tenderness (Table 3).

*TP Loss (%).* This parameter is critical for determining moisture retention and technological efficiency. The NK25 (15.51%) and RK25 (16.66%) groups exhibited the lowest weight losses, indicating an effective maceration treatment with active enzymes at 25 °C. In contrast, the RC (round control) and NC (neck control) samples showed high TP losses (30.43% for RC), highlighting the benefit of enzymatic aging with koji in maintaining juiciness during thermal processing (Table 3).

*pH.* The pH value significantly influences water-holding capacity and tenderness. The recorded pH values ranged between 4.60 and 5.16; this slightly acidic profile is favorable for meat tenderization. A higher pH, such as that observed in the NKS variant (5.16), suggests a stronger buffering effect, possibly due to the soluble proteins released by the Koji enzymes (Table 3).

#### 3.1.2. The Uptake of Curing, Expressible Moisture

Uptake of curing (%). Based on the analysis of six replicates per sample, the RKS and NKS groups exhibited the highest degree of marinade absorption (5.61% and 4.80%, respectively). This demonstrates the efficiency of salt-assisted enzymatic treatment in penetrating the muscle structure (Table 4).

*Expressible moisture.* This parameter reflects water retention within the meat, where higher values may indicate a weaker protein structure. Based on the six replicates per sample, it was observed that RK25 (11.32%) and NK25 (12.16%) had the highest values. This indicates a lower retention of free water and a more permeable structure, likely as an effect of slow aging at a low temperature (Table 4).

The minimum TP loss was observed in the RK25 (16.66 ± 0.02%) and NK25 (15.51 ± 0.01%) groups, whereas the maximum marinade uptake (uptake of curing) was recorded for the RKS (5.61%) and NKS (4.80%) samples (Table 4).

### 3.2. Sensory Characteristics

Regarding sensory characteristics, the highest overall sensory scores perceived by the participants were awarded to the RKS and NKS samples (6.48 and 6.30, respectively). Additionally, the NK25 sample achieved excellent scores for color and juiciness (Table 5).

A comparative analysis between groups R (Round) and N (Neck) revealed significant differences in terms of juiciness and color. The NK25 and NKM samples exhibited significantly higher juiciness compared to RK25 and RKM, respectively. Furthermore, the color of the NK25 sample was rated significantly more favorably than that of RK25 (Table 6).

**Figure 1:**
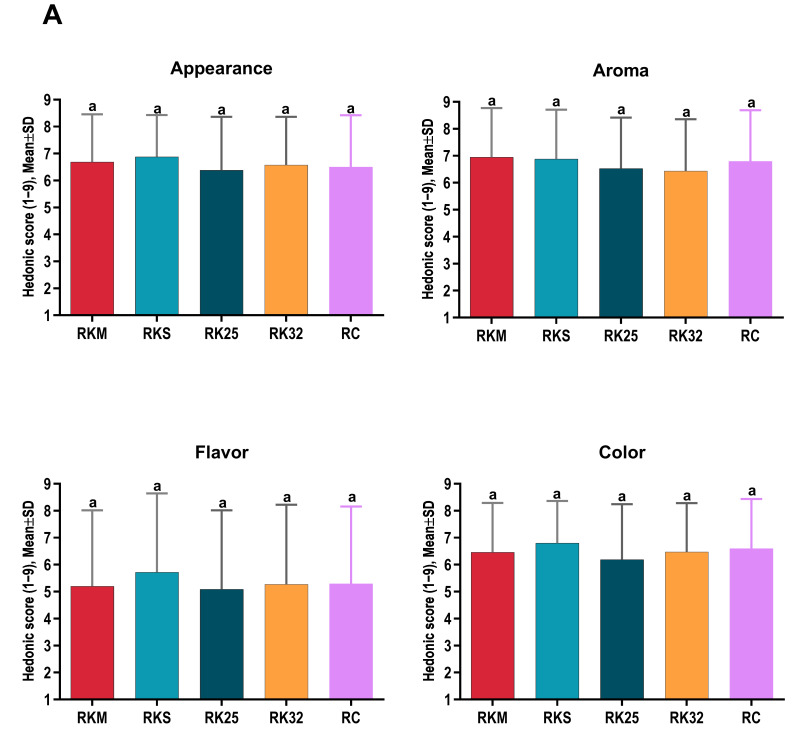

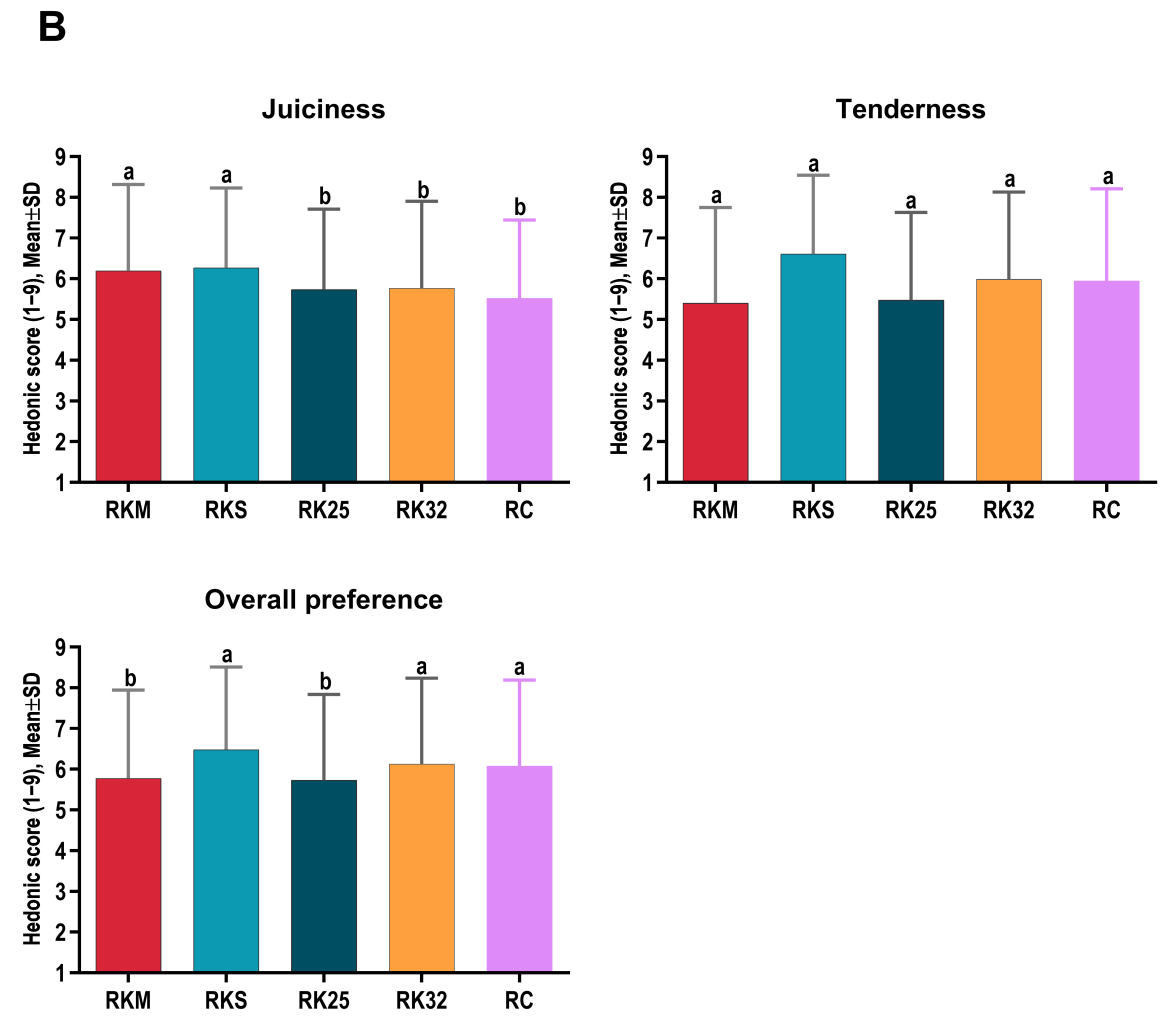
Sensory evaluation of samples. (**A**) Appearance, smell, taste and colour scores of R samples. (**B**) Succulence, tenderness and overall preference scores of R samples. Values represent mean ± SD (n = 6), different letters indicate statistically significant differences (*p* < 0.05).

In the R group, the juiciness of the RKM and RK25 samples was significantly higher compared with the RC control sample (*p* < 0.01 and *p* < 0.001, respectively) (Figure 1).

**Figure 2:**
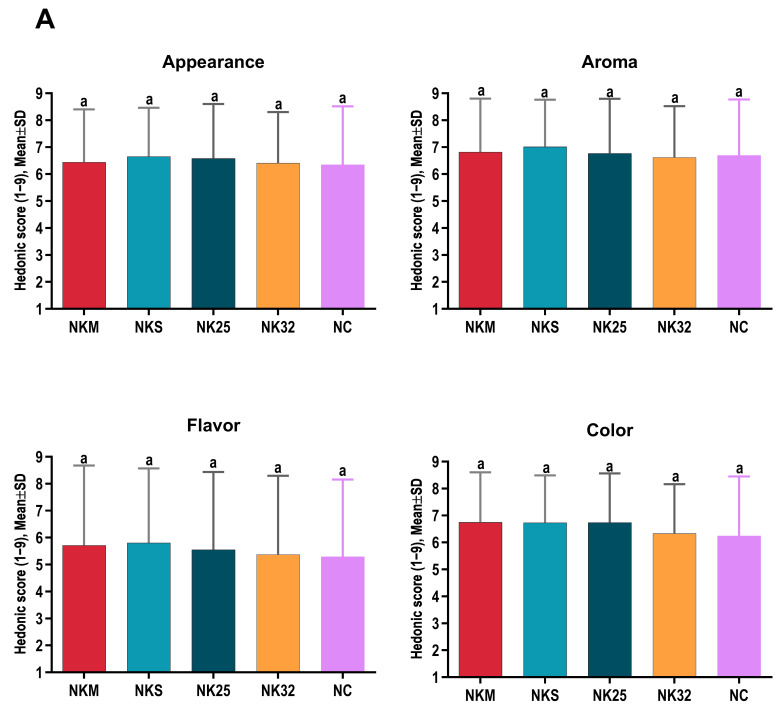

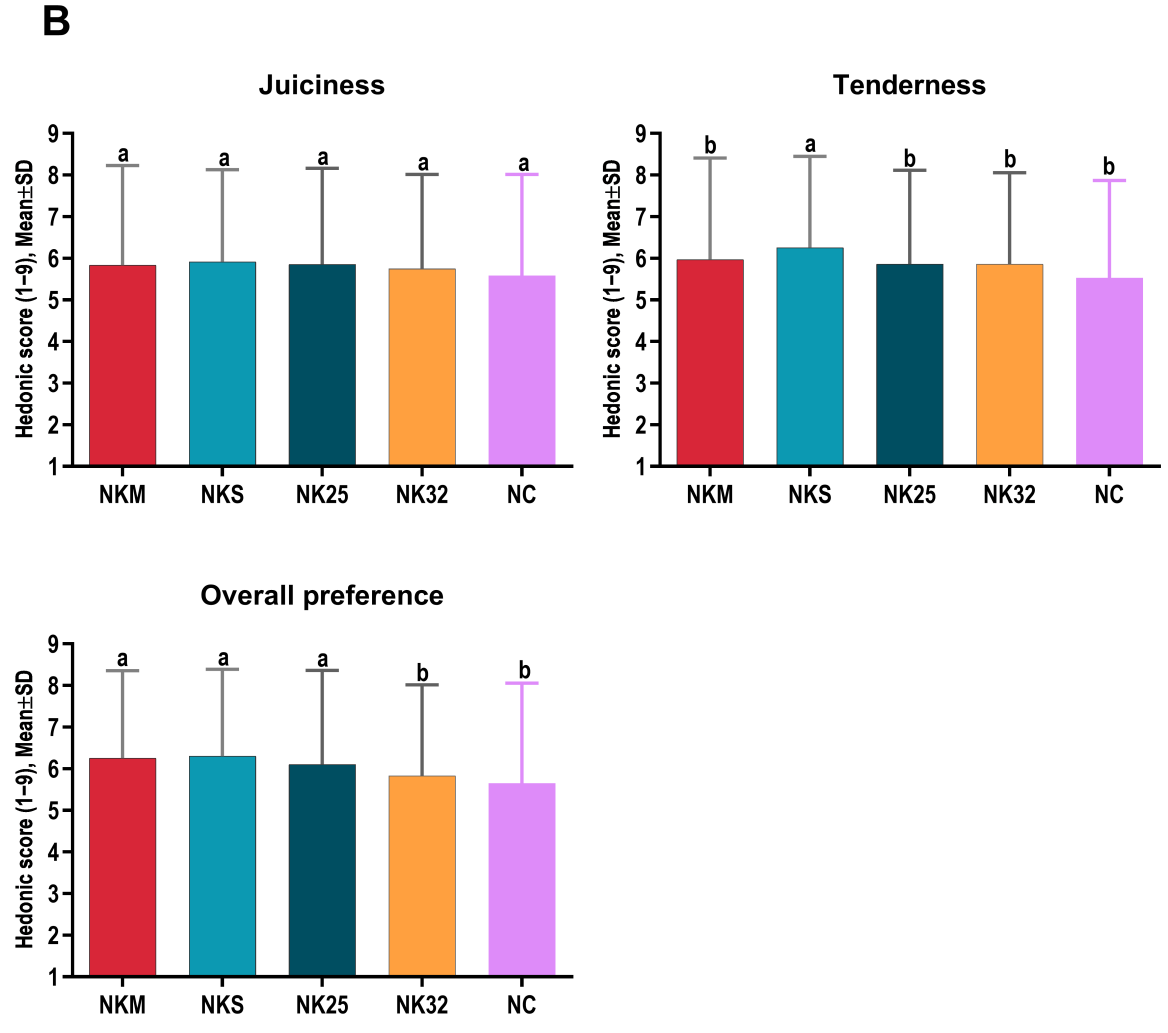
Sensory evaluation of samples. (**A**) Appearance, smell, taste and colour scores of N samples. (**B**) Succulence, tenderness and overall preference scores of N samples. Values represent mean ± SD (n = 6), different letters indicate statistically significant differences (*p* < 0.05).

In the N group, the NKS sample showed a significantly higher mean tenderness compared with the NC control sample (*p* < 0.001) (Figure 2).

### 3.3. Satiating Capacity

Data analysis reveals significant differences between the Taste Group and the Control Group regarding both gustatory perception and the degree of satiety induced by the product.

The results demonstrate that the Taste Group elicited a significantly higher degree of satiety compared to the Control Group. Furthermore, regarding gustatory properties, the data indicate a significant difference between the two samples, with the Taste Group being rated as significantly more pleasant than the Control Group (Table 7).

**Figure 3:**
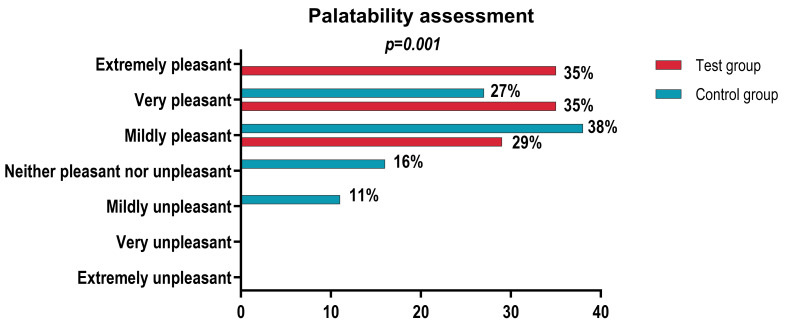
Palatability assessment.

Regarding the gustatory properties, the distribution of responses shows a significantly more favorable appreciation for the Taste group compared to the Control group (*p* = 0.001). None of the participants in the Taste group rated the sample as unpleasant, whereas in the Control group, ratings such as “slightly unpleasant” and “neither pleasant nor unpleasant” were reported. Furthermore, the categories “very pleasant” and “extremely pleasant” were more frequently reported in the Taste group, where 70.6% of the participants ranked the product within these superior levels of appreciation. In contrast, only 27.8% of the Control group reported similar ratings, with no evaluations recorded in the “extremely pleasant” category (Figure 3).

**Figure 4:**
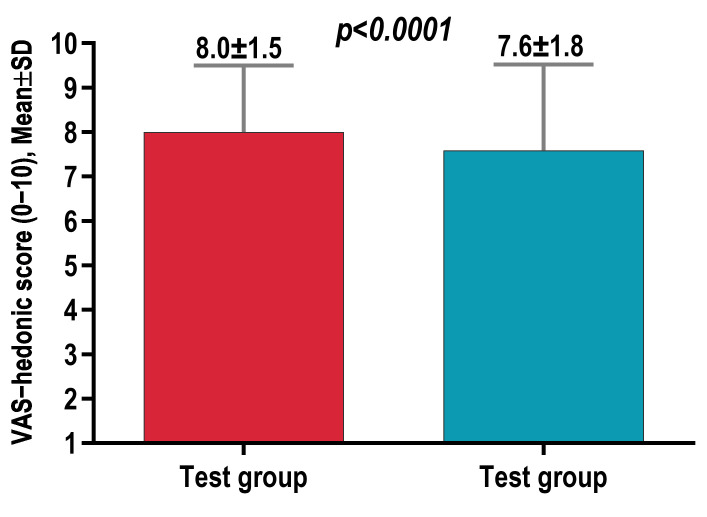
Mean satiety.

Regarding the degree of satiety, the categorical analysis did not reveal significant differences between the groups (*p* = 0.47), with the distribution of responses being relatively similar. However, the majority of participants in both groups rated the product as “quite satiating”, “very satiating”, or “extremely satiating”, with a slight trend toward higher values in the Taste group. This trend is confirmed by the numerical analysis of satiety, where the mean score was significantly higher in the Taste group (8.0 ± 1.5) compared to the Control group (7.61 ± 1.88), with the difference being statistically significant (*p* = 0.0001) (Figure 4). The discrepancy between the categorical analysis (non-significant, *p* = 0.47) and the analysis based on mean satiety scores (significant, *p* = 0.0001) can be explained by differences in statistical sensitivity between the two approaches. The categorical analysis collapses the original 9-point scale into broad classes, reducing variability and statistical power. In contrast, the analysis of mean scores preserves the full resolution of the scale, allowing detection of smaller but consistent differences across treatments. This pattern is common in sensory and perception studies where ordinal-to-categorical transformations may obscure treatment effects.

**Figure 5:**
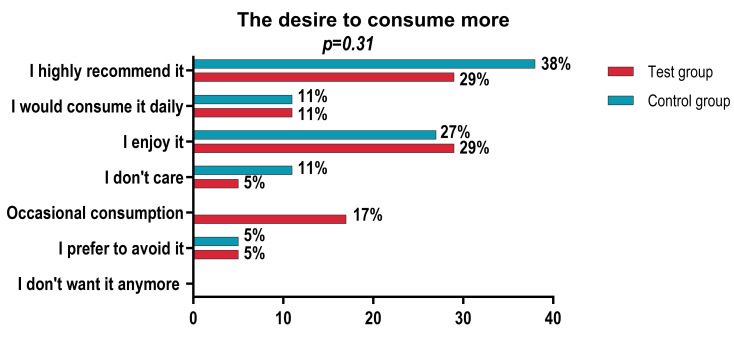
The desire to consume more.

Regarding the future consumption intent, no significant differences were identified between the two groups (*p* = 0.31). In both groups, most participants indicated a positive intention to consume the product (“I enjoy consuming it”, “I would consume it daily” or “I strongly recommend it”), with a comparable proportion between the Taste group and the Control group (Figure 5).

**Figure 6:**
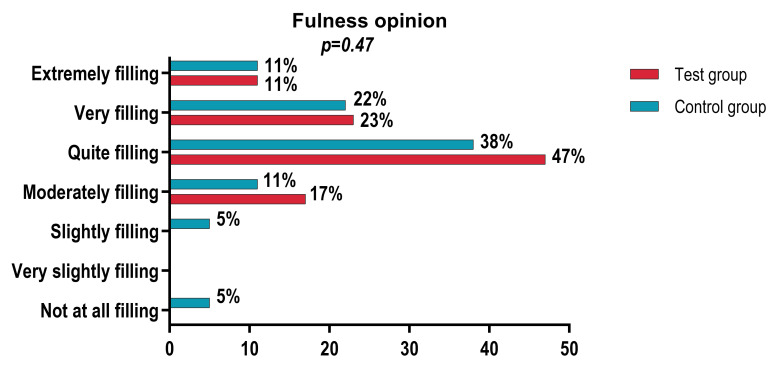
Fullness opinion.

The distribution of responses regarding the perception of fullness was similar between the Test group and the Control group, with no statistically significant differences observed (*p* = 0.47). In both groups, the majority of participants rated the sensation of fullness as pleasant, with the most frequent responses being “slightly pleasant”, “very pleasant”, and “extremely pleasant.” Negative ratings were rare and exclusively reported within the Control group. These results indicate a favorable perception of satiety for both samples (Figure 6).

## 4. Discussion

As evaluating the potential of *Aspergillus oryzae* (Koji) as a biotechnological agent for optimizing meat quality and nutritional value requires high-quality raw materials, Aberdeen Angus beef was selected for this study. This choice was based on the superior standards associated with the livestock rearing ecosystem in the mountain area of Magherani, Mureș County. The organic pastures, characterized by high floristic biodiversity, contribute to a favorable nutritional profile and textural properties suitable for biotechnological processing with *Aspergillus oryzae* (Koji).

### 4.1. Physico-Chemical Properties

The results demonstrate that enzymatic treatment with *Aspergillus oryzae* significantly influences the physicochemical properties of Angus beef, with effects depending on both the Koji activation temperature and the specific anatomical cut analyzed.

The moisture content was higher in the round samples compared to the neck, suggesting distinct muscular structures and an intrinsically lower water-holding capacity in the neck in the absence of enzymatic treatment. This can be explained by differences in muscle tissue structure, intramuscular fat content, and connective tissue, all of which are factors known to influence water retention in meat [14]. Specifically, connective tissue and collagen distribution modulate the degree of contraction and moisture loss during thermal processing, thereby influencing the water-holding capacity (WHC) across different muscles [14].

Among the Koji-treated samples, the NK25 and NK32 variants maintained moisture levels above 55%, suggesting efficient hydration of the muscle matrix. These findings align with the literature regarding the use of *Aspergillus oryzae* and other microbial proteases to enhance the technological quality of meat. Secreted proteases can induce the fragmentation of myofibrillar proteins and collagen, facilitating the release of soluble peptides and creating spaces that favor internal water retention and the juiciness of the final product [4].

The differences between the NK25 and NK32 variants may be attributed to the distinct temperature-dependent enzyme activation profiles. At 25 °C (NK25), enzymatic activation follows a slower kinetic path, allowing proteases to diffuse progressively and uniformly throughout the muscle mass. This gradual distribution limits excessive superficial degradation and helps maintain the structural integrity of the protein matrix. Conversely, activation at higher temperatures (NK32) significantly accelerates proteolytic activity, generating more intense protein hydrolysis. This dynamic can result in more pronounced tenderization, particularly if the aging period exceeds 72 h [15].

TP loss serves as a critical indicator of protein structural stability during thermal treatment; myofibril denaturation and muscle fiber contraction lead to the migration and subsequent loss of water from the meat matrix [16]. The significant reduction in TP loss observed in the Koji-treated samples, particularly in the RK25 and NK25 variants, is compared to the superior water-holding capacity (WHC). This suggests that proteases released by *Aspergillus oryzae* may modify the tertiary structure of proteins, thereby reducing muscle fiber shrinkage and preventing excessive fluid expulsion. These findings align with literature regarding the application of shio-koji to game meat, where such treatments reduced both TP loss and muscular rigidity [16,17]. They are also consistent with studies detailing the effects of Koji treatments on the physicochemical properties of poultry [4,18,19]. In this context, the NK25 variant induced a moderate modification of myofibrillar proteins, sufficient to enhance WHC without compromising the structural cohesion of the muscle fiber. The equilibrium achieved between protein hydrolysis and the maintenance of structural integrity explains both the reduced TP loss and the uniform texture observed experimentally.

The pH values obtained in the koji-treated samples ranged between 4.60 and 5.16, which are slightly lower than the typical pH of beef (5.4–5.7). This decrease may be attributed to the metabolic activity of *Aspergillus oryzae* and Teriyaki sous that lead to the formation of organic acids during the marination process. Additionally, the diffusion of acidic compounds from the marinade and the enzymatic breakdown of muscle proteins may contribute to the observed acidification of the meat matrix. Scientific literature indicates that normal pH values are associated with tenderness and color stability through the enzymatic modulation of myofibrils and sarcoplasmic proteins; conversely, extreme pH values can lead to excessive denaturation or enzyme inactivation, resulting in diminished product quality [20,21,22]. Furthermore, studies investigating Koji treatments identify pH as a key indicator of fermentation-induced physicochemical changes and their influence on meat product quality [1]. Beyond their contribution to flavor development, the peptides and free amino acids generated through *Aspergillus oryzae* proteolysis may also play a significant role in the buffering capacity of the meat system. These low-molecular-weight compounds contain ionizable amino and carboxyl groups capable of stabilizing pH variations during enzymatic aging, thereby contributing to the buffering properties of muscle proteins and their degradation products [23,24]. Therefore, the relatively stable pH values observed in the present study may not only reflect limited acidification but also an enhanced buffering effect resulting from proteolytic activity, as previously reported in enzyme-assisted meat aging systems [25,26,27].

The buffering capacity is intrinsically linked to water-holding capacity and meat tenderness. When muscle pH remains sufficiently distant from the isoelectric point of myofibrillar proteins (~5.1), electrostatic repulsion between protein filaments increases, promoting myofibrillar swelling and improved water retention [23,25]. This mechanism may partly explain the reduced cooking losses and increased tenderness observed in NK25 and RK25 samples, indicating that the improvement of technological properties results from both enzymatic degradation and pH-mediated structural stabilization [26,27].

Uptake of curing was highest in the shio-koji treated samples (RKS and NKS), highlighting the role of salt in facilitating the diffusion of active components into the muscular matrix. NaCl is conventionally used in meat products for its properties during the aging process, often in higher quantities than in sustainable analogues, where such properties may not be required [28]. Studies on NaCl diffusion in muscle tissue show that sodium and chlorine ions diffuse passively into the fibers, influencing the micro- and macro-structure of proteins and the WHC during curing through “salting-in” mechanisms, namely, the solubilization and expansion of myofibrillar filaments [29]. Additionally, recent research suggests that Koji-treated samples benefit from accelerated mass transfer and more efficient penetration of curing agents, due to the synergistic action of salt and protein degradation, which releases soluble materials from the muscle matrix [4].

The activation of enzymes at 25 °C (NK25) exhibits slower dynamics, allowing proteases to penetrate the muscle fiber structure gradually and uniformly. This controlled diffusion minimizes excessive degradation of the superficial layer and helps maintain the integrity of the protein matrix. In contrast, activation at higher temperatures (NK32) rapidly intensifies proteolytic activity, generating accelerated protein hydrolysis. This action can produce more pronounced tenderization, particularly when the aging period exceeds 72 h [30,31]. Compared with previously reported studies on shio-koji and enzyme-assisted meat aging, the magnitude of TP loss reduction and moisture retention observed in this study is comparable or slightly improved despite the use of moderate activation temperatures. Earlier investigations typically reported enhanced tenderness after longer aging periods or higher enzymatic activity levels, whereas the present results suggest that controlled activation at 25 °C may achieve similar technological improvements while preserving muscle structure. This highlights the efficiency of mild enzymatic activation as a potential optimization strategy for accelerated aging processes.

### 4.2. Sensory Characteristics

The sensory analysis confirmed the positive impact of the koji treatment on the perceived quality of beef. The significant differences observed between treated and control samples, particularly in terms of juiciness, tenderness, and color, reflect the physico-chemical modifications previously described, such as the increased water-holding capacity and the structural changes in myofibrillar proteins [4,16].

When comparing anatomical groups, the koji-treated neck samples were evaluated as more succulent, which correlates with their higher water content and reduced TP losses. Within the N group, the tenderness of the NKS sample was significantly higher than that of the control sample, suggesting that the combined effect of *Aspergillus oryzae* enzymes and salt enhances tenderization by degrading structural proteins and reducing muscle-fiber rigidity [17,32,33]. These results are consistent with studies reporting substantial improvements in texture and acceptability in meat matured through alternative fermentative methods, including the use of shio-koji [4].

Furthermore, the observed differences in color can be explained by changes in pH and protein state, factors known to influence the stability of muscular pigments and the visual perception of meat. Literature indicates that maintaining pH within moderate ranges favors myoglobin stability and results in a more attractive color for the consumer [20]. Overall, the sensory data indicate increased acceptability of Koji-treated samples, supporting the potential of this method as a viable alternative to conventional meat aging.

The selection of an internal temperature of 57–59 °C, commonly used in sensory studies of premium beef, was essential to allow for an accurate evaluation of tenderness, juiciness, and aromatic profile. These parameters cannot be adequately assessed at higher temperatures (e.g., 71 °C), where the meat structure is significantly altered [15].

An important methodological consideration involves the properties of the water used in processing and preparation. Parameters such as mineralization, pH, and electrolyte content can influence sensory perception and the water-holding capacity within complex protein matrices. Potential discrepancies between the physicochemical parameters of tap water and bottled/processed waters highlight variabilities in mineral composition and sensory properties. These differences may be relevant for the formulation of maceration solvents and the gustatory perception of food products prepared with them [34,35].

### 4.3. Satiety Capacity

The evaluation of satiety capacity revealed a distinct advantage of the Koji-treated samples compared to the control group. While the categorical distribution of responses did not indicate significant differences between groups, a numerical analysis of the Visual Analogue Scale (VAS) scores demonstrated a significantly higher mean satiety level within the “Taste Group.”

Dietary proteins and the peptides resulting from their digestion interact with enteroendocrine sensors in the intestine, stimulating the secretion of anorexigenic hormones such as cholecystokinin (CCK), glucagon-like peptide-1 (GLP-1), and peptide YY (PYY). These contribute to the sensation of fullness and the subsequent reduction in food intake [36,37,38,39]. Furthermore, the superior gustatory appreciation of the Koji-treated samples may indirectly contribute to the perception of satiety by increasing postprandial satisfaction. These observations support the inclusion of satiety assessment as a relevant tool in the analysis of meat products obtained through innovative fermentative technologies. The increase in satiety scores suggests that aging with *Aspergillus oryzae* (Koji) may offer additional nutritional benefits, contributing to more efficient caloric intake control and body weight maintenance without compromising sensory pleasure.

We posit that Koji treatment can be classified as a form of Accelerated Aging, capable of reproducing a significant portion of the sensory and technological benefits of traditional premium beef aging, while bypassing the prolonged timeframes and economic losses associated with conventional dry-aging processes. In contrast to conventional studies focusing primarily on extended fermentation or high enzymatic intensity, the present study highlights that controlled enzymatic activation combined with anatomical muscle selection can optimize both physicochemical and sensory outcomes within shorter processing times. This integrated approach represents a novel contribution to the development of sustainable accelerated aging technologies.

The results demonstrate that the anatomical region strongly influences the efficiency of enzymatic treatment: the neck/chuck responds far more favorably to the action of *Aspergillus oryzae* proteases than the round, due to its higher collagen and intramuscular fat content elements that are optimally degraded and utilized through controlled proteolysis.

Among the tested variants, NKS and RKS exhibited the most balanced sensory profile, effectively combining tenderness, juiciness, and umami intensity. Concurrently, NK25 and RK25 recorded the lowest TP losses and superior succulence, confirming the efficiency of slow enzymatic activation at 25 °C in maintaining the structural integrity of the muscle fiber.

The high yield and minimal TP losses transform Koji from an experimental concept into a practical and sustainable solution for the meat industry, with genuine potential for integration into modern accelerated aging processes.

## 5. Conclusions

The results of this study demonstrate that the use of *Aspergillus oryzae* (Koji) as a biotechnological agent represents an effective strategy for enhancing the quality of Angus beef. The enzymatic treatment resulted in superior water retention, reduced TP losses, and more efficient absorption of the maceration mixture, indicating favorable structural modifications within the meat matrix.

Sensory analysis revealed significant improvements in tenderness, juiciness, and overall acceptability, particularly in the samples treated with shio-koji. Furthermore, satiety testing indicated that the Koji-aged variant induces a higher level of perceived fullness compared to traditional marination, without negatively affecting the desire for repeated consumption.

Overall, the data support the potential of Koji as a natural, sustainable, and innovative solution for optimizing red meat quality. These findings have relevant applications in the food industry, improving sensory and technological properties.

## Figures and Tables

**Table 1:** Sample Coding and Experimental Design.

Beef Round Samples (Group R)	Beef Neck Samples (Group N)	Sample Preparation
**Round** **-K** **oji-Mix-Shiitake (RKM)**	**Neck-Koji-Mix-Shiitake (NKM)**	• *Aspergillus oryzae* was obtained by incubation at 32–33 °C on a substrate of cooked and cooled rice grains. • After 4 days of incubation and fungal proliferation, the product was dried to a moisture content of 3–5% and finely ground. • The enzymatic mix was prepared from 70% Koji powder and 30% shiitake (Lentinula edodes) powder, both dried and finely milled, then manually blended. • The powder mixture was hydrated for 3–5 h at 22 °C, forming a homogeneous paste. • The paste was applied uniformly in a thicker layer onto both the round and neck cuts. • The meat was kept under refrigeration for 72 h to allow enzymatic action. • At the end of the process, the excess enzymatic mixture was removed.
**Round-Shio-Koji (RKS)**	**Neck-Shio-Koji (NKS)**	• *Aspergillus oryzae* was obtained by incubation at 32–33 °C on a substrate of cooked and cooled rice grains. • After 4 days of incubation, the product was dried to a moisture content of 3–5%, finely ground, and mixed with fine non-iodized salt (25%). • The Koji mixture was hydrated for 3 h at 22 °C to activate the enzymes and obtain a homogeneous paste. • The resulting paste was applied uniformly and thoroughly onto both the neck and round cuts, ensuring complete coverage. • The enzymatic maturation process was carried out under refrigeration for 72 h. • At the end of the process, the excess Koji was removed by rinsing.
**RK32**	**NK32**	• *Aspergillus oryzae* was obtained by incubation at 32–33 °C on a substrate of cooked and cooled rice grains. • After 4 days of incubation, the product was dried to a moisture content of 3–5% and finely ground. • The resulting powder underwent controlled hydration at 32–33 °C to activate the thermophilic enzymes produced by *Aspergillus oryzae*, enhancing protease activity and accelerating the hydrolysis of protein bonds. • The mixture was applied in two thin, layered coatings onto both the neck and round cuts, with a 30 min interval between applications to ensure optimal absorption. • The enzymatic maturation process was carried out under extended refrigeration for 72 h. • At the end of the process, the excess enzymatic material was removed.
**RK25**	**NK25**	• *Aspergillus oryzae* was obtained by incubation at 25–26 °C on a substrate of cooked and cooled rice grains. • After 4 days of incubation and fungal proliferation, the product was dried to a moisture content of 3–5% and finely ground. • The Koji powder was hydrated with sterilized water for 5 h at 22 °C to activate the enzymes; the NK25 variant relied on lower incubation temperature and prolonged hydration time, resulting in slower enzymatic activation. • The mixture was applied uniformly onto the surface of the beef cuts (neck and round) in a single even layer. • Enzymatic maturation was carried out by refrigerating the treated meat for 72 h at 2–4 °C, ensuring a slow and balanced enzymatic action. • At the end of the process, the excess Koji was removed.
**RC**	**NC**	Marination with Teriyaki sauce

**Table 2:** Physicochemical Parameters (mean ± SD).

Parameter	Round–Koji (Mean ± SD)	Round–Control (RC) (Mean ± SD)	Neck–Koji (Mean ± SD)	Neck–Control (NC) (Mean ± SD)
Water content (%)	55.22 ± 3.47 ^a^	49.71 ± 0.45 ^b^	55.27 ± 2.00 ^a^	45.74 ± 0.45 ^c^
Cooking loss (%)	24.85 ± 6.39 ^a^	30.43 ± 0.60 ^b^	22.89 ± 9.18 ^a^	35.00 ± 0.55 ^c^
pH	4.88 ± 0.13 ^a^	4.64 ± 0.10 ^b^	5.04 ± 0.13 ^c^	4.60 ± 0.10 ^b^
Enzymatic uptake (%) *	4.64 ± 0.80 ^a^	−0.60 ± 0.10 ^b^	4.07 ± 0.70 ^a^	−0.50 ± 0.10 ^b^
Expressible moisture (%)	5.29 ± 3.80 ^a^	0.91 ± 0.06 ^b^	5.41 ± 4.00 ^a^	1.02 ± 0.07 ^b^

Different superscript letters indicate statistically significant differences between groups (one-way ANOVA followed by Bonferroni post hoc test, *p* < 0.05). Values are expressed as mean ± SD (n = 6). * The negative uptake values observed in the control samples (RC and NC) indicate a slight loss of moisture/weight during the conventional Teriyaki marination process, in contrast to the Koji-treated samples, where an active absorption of the enzymatic mixture occurred.

**Table 3:** Water content, weight of meat before and after TP, and pH (mean ± SD).

Sample n = 6	Water Content (%) (Mean ± SD)	TP Loss (%) (Mean ± SD)	pH (Mean ± SD)
RKM	54.08 ± 0.2	25 ± 0.3	4.92 ± 0.1
RKS	59.47 ± 0.3	25.8 ± 0.4	4.85 ± 0.1
RK25	50.99 ±0.3	16.66 ± 0.45	5.03 ± 0.1
RK32	56.33 ± 0.25	31.94 ± 0.45	4.72 ± 0.1
RC	49.71 ± 0.45	30.43 ± 0.6	4.64 ± 0.1
NKM	52.15 ± 0.35	38.23 ± 0.6	5.13 ± 0.1
NKS	56.98 ± 0.3	19.64 ± 0.45	5.16 ± 0.1
NK25	55.48 ± 0.2	15.51 ± 0.3	4.9 ± 0.1
NK32	56.48 ± 0.25	18.18 ± 0.45	4.96 ± 0.1
NC	45.74 ± 0.45	35 ± 0.55	4.60 ± 0.1

**Table 4:** The uptake of curing. Expressible moisture (mean ± SD).

Sample n = 6	Uptake of Curing (%) (Mean ± SD)	Expressible Moisture (%) (Mean ± SD)
**RKM**	4.65 ± 0.01 ^a^	2.37 ± 0.08 ^a^
**RKS**	5.61 ± 0.03 ^b^	4.51 ± 0.08 ^b^
**RK25**	4.63 ± 0.03 ^a^	11.32 ± 0.13 ^c^
**RK32**	3.66 ± 0.03 ^c^	2.94 ± 0.07 ^a^
**RC**	−0.60 ± 0.10 ^d^	0.91 ± 0.06 ^d^
**NKM**	3.10 ± 0.02 ^a^	4.14 ± 0.14 ^a^
**NKS**	4.80 ± 0.03 ^b^	3.73 ± 0.08 ^a^
**NK25**	3.76 ± 0.02 ^c^	12.16 ± 0.15 ^b^
**NK32**	4.60 ± 0.03 ^b^	1.62 ± 0.08 ^c^
**NC**	−0.50 ± 0.10 ^d^	1.02 ± 0.07 ^c^

Different superscript letters within each anatomical region indicate statistically significant differences between treatments (one-way ANOVA followed by Bonferroni post hoc test, *p* < 0.05). Values are expressed as mean ± SD (n = 6).

**Table 5:** Sensorial scores (mean ± SD).

Parameter	Round–Koji (Mean ± SD)	Round Control (Mean ± SD)	Neck–Koji (Mean ± SD)	Neck Control (Mean ± SD)
Appearance	6.63 ± 0.12 ^a^	6.50 ± 0.10 ^a^	6.52 ± 0.11 ^a^	6.35 ± 0.10 ^a^
Aroma	6.70 ± 0.10 ^a^	6.79 ± 0.12 ^a^	6.81 ± 0.11 ^a^	6.70 ± 0.10 ^a^
Flavor	5.34 ± 0.15 ^a^	5.29 ± 0.14 ^a^	5.62 ± 0.13 ^b^	5.30 ± 0.12 ^a^
Color	6.48 ± 0.11 ^a^	6.60 ± 0.10 ^a^	6.64 ± 0.12 ^a^	6.24 ± 0.11 ^b^
Juiciness	5.99 ± 0.14 ^a^	5.52 ± 0.12 ^b^	5.84 ± 0.13 ^a^	5.58 ± 0.12 ^b^
Tenderness	5.87 ± 0.13 ^a^	5.95 ± 0.12 ^a^	5.99 ± 0.14 ^a^	5.53 ± 0.12 ^b^
Overall preference	6.03 ± 0.12 ^a^	6.08 ± 0.11 ^a^	6.12 ± 0.12 ^a^	5.65 ± 0.11 ^b^

Different superscript letters within each anatomical region indicate statistically significant differences between treatments (one-way ANOVA followed by Bonferroni post-hoc test, *p* < 0.05). Values are expressed as mean ± SD (n = 6).

**Table 6:** Hedonic rating of beef with different curing treatments (mean ± SD).

Sample (n = 6)	Appearance (Mean ± SD)	Aroma (Mean ± SD)	Flavor (Mean ± SD)	Color (Mean ± SD)	Juiciness (Mean ± SD)	Tenderness (Mean ± SD)	Overall Preference (Mean ± SD)
RKM	6.69 ± 1.76 ^a^	6.95 ± 1.81 ^a^	5.28 ± 2.80 ^a^	6.46 ± 1.83 ^a^	6.19 ± 2.12 ^a^	5.40 ± 2.34 ^a^	5.78 ± 2.16 ^b^
RKS	6.88 ± 1.55 ^a^	6.88 ± 1.83 ^a^	5.72 ± 2.91 ^a^	6.80 ± 1.56 ^a^	6.27 ± 1.95 ^a^	6.61 ± 1.93 ^a^	6.48 ± 2.02 ^a^
RK25	6.38 ± 1.97 ^a^	6.53 ± 1.88 ^a^	5.09 ± 2.92 ^a^	6.19 ± 2.05 ^a^	5.73 ± 1.97 ^b^	5.48 ± 2.15 ^a^	5.73 ± 2.10 ^b^
RK32	6.58 ± 1.78 ^a^	6.44 ± 1.92 ^a^	5.28 ± 2.94 ^a^	6.48 ± 1.81 ^a^	5.77 ± 2.13 ^b^	5.99 ± 2.14 ^a^	6.13 ± 2.11 ^a^
RC	6.50 ± 1.92 ^a^	6.79 ± 1.89 ^a^	5.29 ± 2.86 ^a^	6.60 ± 1.83 ^a^	5.52 ± 1.91 ^b^	5.95 ± 2.25 ^a^	6.08 ± 2.10 ^a^
NKM	6.44 ± 1.96 ^a^	6.82 ± 1.98 ^a^	5.72 ± 2.95 ^a^	6.75 ± 1.85 ^a^	5.84 ± 2.39 ^a^	5.97 ± 2.43 ^b^	6.26 ± 2.09 ^a^
NKS	6.66 ± 1.81 ^a^	7.02 ± 1.74 ^a^	5.81 ± 2.76 ^a^	6.74 ± 1.75 ^a^	5.92 ± 2.21 ^a^	6.26 ± 2.19 ^a^	6.30 ± 2.08 ^a^
NK25	6.58 ± 2.01 ^a^	6.77 ± 2.02 ^a^	5.56 ± 2.87 ^a^	6.74 ± 1.81 ^a^	5.86 ± 2.30 ^a^	5.86 ± 2.25 ^b^	6.10 ± 2.25 ^a^
NK32	6.41 ± 1.89 ^a^	6.62 ± 1.89 ^a^	5.38 ± 2.92 ^a^	6.34 ± 1.82 ^a^	5.75 ± 2.26 ^a^	5.86 ± 2.19 ^b^	5.83 ± 2.18 ^b^
NC	6.35 ± 2.16 ^a^	6.70 ± 2.07 ^a^	5.30 ± 2.86 ^a^	6.24 ± 2.21 ^a^	5.58 ± 2.43 ^a^	5.53 ± 2.34 ^b^	5.65 ± 2.40 ^b^

Different superscript letters within each anatomical region indicate statistically significant differences between treatments (one-way ANOVA followed by Bonferroni post-hoc test, *p* < 0.05). Values are expressed as mean ± SD (n = 6).

**Table 7:** Statistical results for determining the degree of satiety.

Parameters	Taste Group		Control Group		
	n	%	n	%	*p* Value
**T** **aste**					
Extremely unpleasant	0	0	0	0	0.001
Very unpleasant	0	0	0	0	
Slightly unpleasant	0	0	2	11.1	
Neither pleasant nor unpleasant	0	0	3	16.7	
Slightly pleasant	5	29.4	7	38.9	
Very pleasant	6	35.3	5	27.8	
Extremely pleasant	6	35.3	0	0	
**Satiety**					
Not satiating at all	0	0	1	5.6	0.47
Very slightly satiating	0	0	0	0	
Slightly satiating	0	0	1	5.6	
Moderately satiating	3	17.6	2	11.1	
Fairly satiating	8	47.1	7	38.9	
Very satiating	4	23.5	4	22.2	
Extremely satiating	2	11.8	2	11.1	
**Desire to consume again**					
I do not want any more	0	0	0	0	0.31
I prefer to avoid it	1	5.9	1	5.6	
Occasional consumption	3	17.6	0	0	
I am indifferent	1	5.9	2	11.6	
I enjoy consuming it	5	29.4	5	27.8	
I would consume it daily	2	11.8	2	11.1	
I strongly recommend it	5	29.4	7	38.9	
**Satiety score** **- Numeric, mean ± SD**	8.0 ± 1.5		7.61 ± 1.88		0.0001

## Data Availability

The study data is available upon request.

## References

[1] Simrén M., Barbara G., Flint H.J., Spiegel B.M.R., Spiller R.C., Vanner S., Verdu E.F., Whorwell P.J., Zoetendal E.G. (2013). Intestinal microbiota in functional bowel disorders: A Rome foundation report. Gut.

[2] Ruta F., Pribac M., Mardale E., Suciu S., Maior R., Bogdan S., Avram C. (2024). Associations between Gut Microbiota Dysbiosis and Other Risk Factors in Women with a History of Urinary Tract Infections. Nutrients.

[3] Sullivan G.A., Calkins C.R. (2010). Application of exogenous enzymes to beef muscle of high- and low-connective tissue. Meat Sci..

[4] Jeong J., Jeon S., Lee J., Lee M.-Y., Lee K.-H., Song C.-K., Choi M.-J. (2023). The Effect of Fermented Grains (Koji) on Physicochemical and Sensory Characteristics of Chicken Breasts. Foods.

[5] Song P., Zhang X., Wang S., Xu W., Wang F., Fu R., Wei F. (2023). Microbial proteases and their applications. Front. Microbiol..

[6] Yamashita H. (2021). Koji Starter and Koji World in Japan. J. Fungi.

[7] Machida M., Yamada O., Gomi K. (2008). *Aspergillus oryzae*: A Model Filamentous Fungus for Industrial Application. Trends Biotechnol..

[8] Oda K. (2012). New Families of Carboxyl Peptidases: Structural and Functional Diversity. Appl. Microbiol. Biotechnol..

[9] de Castro R.J.S., Sato H.H. (2014). Protease from *Aspergillus oryzae*: Biochemical Characterization and Application as a Potential Biocatalyst for Production of Protein Hydrolysates with Antioxidant Activities. J. Food Process..

[10] Association of Official Analytical Chemists (2019). Official Methods of Analysis of AOAC International.

[11] Flint A., Raben A., Blundell J.E., Astrup A. (2000). Reproducibility, power and validity of visual analogue scales in assessment of appetite sensations. Int. J. Obes..

[12] Chambers L., McCrickerd K., Yeomans M.R. (2015). Optimising foods for satiety. Trends Food Sci. Technol..

[13] Avram C., Mărușteri M. (2022). Normality assessment, few paradigms and use cases. Rom. J. Lab. Med..

[14] Wang J., Yang P., Han D., Huang F., Li X., Song Y., Wang H., Liu J., Zheng J., Zhang C. (2022). Role of Intramuscular Connective Tissue in Water Holding Capacity of Porcine Muscles. Foods.

[15] Torun M., Khan M., Rahman M., Sadakuzzaman M., Hashem M. (2023). Influence of degree of doneness temperature on the sensory, physiochemical, nutritional, and microbial properties of beef. Meat Res..

[16] Honikel K.O. (1998). Water-holding capacity of meat-A review of the causes of water loss. Meat Sci..

[17] Hiemori-Kondo M., Ueta R., Nagao K. (2022). Improving deer meat palatability by treatment with ginger, shio-koji, and miso. Meat Sci..

[18] Chang H.-C., Kim D.-H. (2014). Enzymatic characteristics of *Aspergillus oryzae* in Koji fermentation and its applications. J. Appl. Microbiol..

[19] Liu Y., Liu C., Wang J., Zheng F., Li Q., Niu C. (2025). Biochemical characterization of proteolytic enzymes from *Aspergillus oryzae*. Int. J. Biol. Macromol..

[20] Sristi P., Das N., Akhter A., Kaniya N., Hashem M. (2025). Relation among meat pH, color and tenderness—A review. Meat Res..

[21] Jankowiak H., Cebulska A., Bocian M. (2021). The relationship between acidification (pH) and meat quality parameters. Eur. Food Res. Technol..

[22] Li P., Wang T., Mao Y., Zhang Y., Niu L., Liang R., Zhu L., Luo X. (2014). Effect of ultimate pH on postmortem myofibrillar protein degradation. Sci. World J..

[23] Puolanne E., Halonen M. (2010). Theoretical Aspects of Water-Holding in Meat. Meat Sci..

[24] Toldrá F., Reig M. (2011). Innovations for Healthier Processed Meats. Trends Food Sci. Technol..

[25] Huff-Lonergan E., Lonergan S.M. (2005). Mechanisms of Water-Holding Capacity of Meat: The Role of Postmortem Biochemical and Structural Changes. Meat Sci..

[26] Mora L., Toldrá F. (2023). Advanced enzymatic hydrolysis of food proteins for the production of bioactive peptides. Curr. Opin. Food Sci..

[27] Toldrá F., Gallego M., Reig M., Aristoy M.-C.L. (2020). Bioactive peptides generated in the processing of dry-cured ham. Food Chem..

[28] Filip C., Kovács B., Casian T., Miklos A., Pop P.N., Istrate T.I., Hodoroga V., Tero-Vescan A. (2025). Comparative analysis of the nutritional quality of plant-based and traditional meat products. Front. Nutr..

[29] Graiver S.L., Marchello M., Kessler H.G. (2006). Diffusion and interaction of salts in meat systems. J. Food Eng..

[30] Su N.-W., Wang M.-L., Kwok K.-F., Lee M.-H. (2005). Effects of temperature and sodium chloride concentration on the activities of proteases and amylases in soy sauce koji. J. Agric. Food Chem..

[31] Bechman A., Phillips R.D., Chen J. (2012). Changes in selected physical property and enzyme activity of rice and barley koji during fermentation and storage. J. Food Sci..

[32] Xiong Y.L. (2005). Role of myofibrillar proteins in water-binding in brine-enhanced meats. Food Res. Int..

[33] Toldrá F., Flores M. (1998). Role of muscle proteases and lipases in flavor development. Crit. Rev. Food Sci. Nutr..

[34] Ruta F., Avram C., Maior R. (2023). Physico-Chemical and Microbiological Differences between Mains and Bottled Water. Int. J. Environ. Res. Public Health.

[35] Anderson G.H., Moore S.E., Ziesel S., Woodend D. (2004). Dietary proteins in regulation of food intake. J. Nutr..

[36] Potier M., Darcel N., Tomé D. (2009). Protein, amino acids and control of food intake. Curr. Opin. Clin. Nutr. Metab. Care.

[37] Ignot-Gutiérrez A., Serena-Romero G., Guajardo-Flores D., Alvarado-Olivarez M., Martínez A.J., Cruz-Huerta E. (2024). Proteins and Peptides from Food Sources with Effect on Satiety and Their Role as Anti-Obesity Agents: A Narrative Review. Nutrients.

[38] Braden M.L., Gwin J.A., Leidy H.J. (2023). Protein source influences acute appetite and satiety. J. Nutr..

[39] Watson A.W., Brooks A., Moore L., Barley S., Holliday A. (2025). The effect of consuming different dietary protein sources at breakfast upon self-rated satiety, peptide YY, glucagon like peptide-1, and subsequent food intake in young and older adults. Eur. J. Nutr..

